# Bloodstream infection following 217 consecutive systemic-enteric drained pancreas transplants

**DOI:** 10.1186/1471-2334-6-127

**Published:** 2006-08-08

**Authors:** Natalie Berger, Sigmund Guggenbichler, Wolfgang Steurer, Christian Margreiter, Gert Mayer, Reinhold Kafka, Walter Mark, Alexander R Rosenkranz, Raimund Margreiter, Hugo Bonatti

**Affiliations:** 1 Department of General and Transplant Surgery, Medical University, Innsbruck, Austria; 2 Clinical Division of Nephrology, Medical University, Innsbruck, Austria; 3 Department of Surgery, Mayo Clinic, Jacksonville, Florida, USA

## Abstract

**Background:**

Combined kidney pancreas transplantation (PTx) evolved as excellent treatment for diabetic nephropathy. Infections remain common and serious complications.

**Methods:**

217 consecutive enteric drained PTxs performed from 1997 to 2004 were retrospectively analyzed with regard to bloodstream infection. Immunosuppression consisted of antithymocyteglobuline induction, tacrolimus, mycophenolic acid and steroids for the majority of cases. Standard perioperative antimicrobial prophylaxis consisted of pipercillin/tazobactam in combination with ciprofloxacin and fluconazole.

**Results:**

One year patient, pancreas and kidney graft survival were 96.4%, 88.5% and 94.8%, surgical complication rate was 35%, rejection rate 30% and rate of infection 59%. In total 46 sepsis episodes were diagnosed in 35 patients (16%) with a median onset on day 12 (range 1–45) post transplant. Sepsis source was intraabdominal infection (IAI) (n = 21), a contaminated central venous line (n = 10), wound infection (n = 5), urinary tract infection (n = 2) and graft transmitted (n = 2). Nine patients (4%) experienced multiple episodes of sepsis. Overall 65 pathogens (IAI sepsis 39, line sepsis 15, others 11) were isolated from blood. Gram positive cocci accounted for 50 isolates (77%): Coagulase negative staphylococci (n = 28, i.e. 43%) (nine multi-resistant), Staphylococcus aureus (n = 11, i.e. 17%) (four multi-resistant), enterococci (n = 9, i.e. 14%) (one E. faecium). Gram negative rods were cultured in twelve cases (18%). Patients with blood borne infection had a two year pancreas graft survival of 76.5% versus 89.4% for those without sepsis (p = 0.036), patient survival was not affected.

**Conclusion:**

Sepsis remains a serious complication after PTx with significantly reduced pancreas graft, but not patient survival. The most common source is IAI.

## Background

Simultaneous pancreas kidney transplantation (SPK) has evolved as an effective treatment modality for patients with end stage diabetic nephropathy with excellent patient, pancreas and kidney graft survival [1]. This improved outcome has mainly been achieved by a reduction in technical failures and more powerful immunosuppression with agents such as Tacrolimus, Sirolimus and mycophenolic acid (MMF) [2-5]. However, these more powerful new immunosuppressive protocols may lead to an increase in infectious complications [6,7]. Intraabdominal infection (IAI) has been shown to be frequently associated with blood stream infection and IAI with sepsis frequently leads to graft loss [6]. Blood stream infections (BSI) seem to influence both graft and patient survival [8,9]. Systemic infections may originate from intraabdominal, renal or pulmonary origin or contaminated intravenous lines [10]. The spectrum of pathogens isolated from blood culture or from catheter tips differs between line sepsis and sepsis from other sources [11]. Coagulase negative staphylococci seem to be the predominant organisms causing line sepsis, whereas the spectrum of sepsis deriving from other sources is much more diverse [12]. Long term vascular access devices such as dialysis catheters have a particular high risk to become contaminated [13]. The diagnosis of sepsis post transplant may be difficult, because immunosuppressive agents influence parameters, which are indicative for inflammation such as the levels of C-reactive protein (CRP) and the white blood count (WBC) and moreover, these parameters can be elevated during graft rejection [14]. Furthermore, during the early post transplant period fever can be caused by a multitude of events and due to the application of steroids patients will not develop pyrexia, even in the case of proven infection. Therefore, a positive blood culture must be considered a serious event and prompt further investigations even if patients do not present with any clinical signs of infection. It seems to be justified to monitor tips of indwelling devices in order to diagnose infection at the earliest possible time point.

The more physiologic enteric exocrine drainage (ED) for pancreas transplantation is now widely accepted, but it is associated with a higher risk for intraabdominal infections and subsequent sepsis as compared to bladder drainage [15,16]. Due to the underlying disease, the necessary central venous access and the high prevalence of IAI, pancreatic recipients are at high risk to develop systemic infections. Data on sepsis in pancreatic recipients are scarce, in particular with regard to cohorts receiving more powerful immunosuppression, patients with advanced age or those suffering from type II diabetes mellitus, and when using grafts from extended criteria donors. Also it is not clearly defined, if retransplantation is associated with a higher risk for sepsis.

In this study of 217 consecutive enteric/systemic drained pancreas transplants all cases of bloodstream infections were retrospectively analyzed. Risk factors for the development of sepsis were investigated and the impact of sepsis on patient, renal and pancreas graft survival with regard to the different sources was determined.

## Methods

This is a consecutive cohort study including all patients who underwent pancreas transplantation at a single center during a seven year period.

### Donor and recipient demographics

Between March 1997 and October 2004, 217 consecutive patients (m/f = 133/84) with a median age of 41.9 years (range 22.4–62.5) underwent pancreas transplantation at our center: 89% together with a kidney from the same donor, which was placed retroperitoneally in the left iliac fossa (SPK). Twenty patients received a pancreas graft sometime after kidney transplantation (PAK), and three patients a pancreas transplant alone (PTA). In 24 instances the pancreas (three times as SPK and 21 times after a previous kidney or kidney/pancreas transplant) and in three cases the kidney were retransplants. Fourteen patients suffered from type II diabetes mellitus. Mean donor age was 29 years (range 10–54). Mean cold ischemia time for the kidney transplants was 11.7 ± 3.3 hrs, and for the pancreas transplants 13.1 ± 3.2 hrs. All pancreatic grafts were revascularized in an end-to-side fashion with the inferior vena cava and via a donor iliac Y-graft with the right common iliac artery. Exocrine drainage was completed as S/S duodeno-jejunostomy except for two duodenocystostomies, in the last 153 instances using a stapling device [3].

### Immunosuppression

213 patients received a single shot antithymocyteglobuline (ATG) (Thymoglobulin, Sangstat or ATG Fresenius) at begin of surgery or a three days course at a lower dosage. One patient did not receive antibody induction during pancreas retransplantation, and three patients received basiliximab (Simulect ^®^, Novartis, Switzerland) or daclizumab (Zenapax ^® ^Roche, Switzerland). MMF (Roche, Switzerland) was given at a dose of 1 g twice daily orally. A rapid steroid tapering regimen was applied starting with 500 mg Methylprednisolon intraoperatively to reach a dose of 25 mg at the end of the first p.o. week and further reduction to a maintenance daily dose of 5 mg. 198 patients received oral Tacrolimus (Tac, Fujisawa, Munich, Germany) at 0.08 mg/kg/day twice daily, starting six hours after revascularization. The Tac dose was adjusted to achieve whole blood trough levels of 10–12 ng/ml for the first three months after transplantation, 8–10 ng/ml for 6–12 months and 6–8 ng/ml thereafter. A total of 11 patients were given Cyclosporin A (CsA, Novartis, Basle, Switzerland) instead of Tac (targeted trough levels of 200–250 ng/dl) and another 19 received Sirolimus (Whyeth, USA) with targeted trough levels of 6–10 ng/ml in combination with Tac as initial immunosuppression. Four patients were switched from Tac to CsA and another four from CsA to Tac. CsA was only used during the early study period (1997–1998) as part of the EURO SPK I trial and Sirolimus was introduced 2002 as part of the EURO SPK II trial.

### Perioperative antimicrobial prophylaxis

A total of 30 patients transplanted between 1997 and 1998 received Amoxicillin/Clavulanic acid 3 × 2, 2 g and 157 patients transplanted between 1998 and 2004 received Piperacillin/Tazobactam 3 × 4, 5 g for 48–72 hours as perioperative systemic antimicrobial prophylaxis and 30 patients received other antimicrobials in case of Penicillin allergy. Quinolones were added as prophylactic agents in the last 106 cases. A total of 168 patients in addition received Fluconazol (400 mg daily) for seven days. Prophylaxis for CMV infection was used in 31 CMV seronegative patients receiving a CMV positive graft (Ganciclovir 2 × 5 mg/kg/day for 10 days, followed by 3000 mg orally for 2 months or recently Valganciclovir 2 × 450 mg). All other patients were monitored on a weekly basis and treated preemptively if tested positive for CMV replication.

### Bacterial/fungal monitoring

Immediately before transplantation a sample of preservation solution and parts of the donor ureter, ascites and urine from the recipient were taken. Post-transplant tips of removed urinary as well as intravascular catheters as well as tips of all intraabdominal drains were sent for microbiological investigation. During ICU stay fluids from thoracic or intraabdominal drains and tracheal or bronchial secretions were taken every day. Sputa, tracheal aspirations, bronchoalveolar lavages, aspiration fluids, wound swabs, cerebrospinal fluid, blood and biopsies were sent whenever indicated. Blood cultures were taken in cases of fever (higher 38 centigrade): two to four consecutive samples within four hours. These samples were cultured aerobically as well as anaerobically for seven days before being reported out negative.

### Definition of sepsis

Bacterial/fungal infection was assumed when a positive culture and clinical signs and/or laboratory parameters requiring antibiotic treatment. Positive surveillance cultures without clinical symptoms were considered colonization. In all cases of sepsis in this series a causing organism could be cultured from blood or a catheter tip or both. Positive blood culture or isolation of an organism from blood without clinical symptoms or laboratory abnormalities were not considered sepsis but prompted further investigations. Clinical signs included fever higher 38 centigrade; laboratory indicators were leucocytosis (> 10000 WBC) or leucopenia (< 3000 WBC) and elevated C-reactive protein (CRP). For coagulase negative staphylococci, sepsis was only considered if two isolates from blood cultures or one positive blood culture with a second isolate from a contaminated line or other source of sepsis were present.

### Viral monitoring

The donor CMV status was recorded from the report of the donor center and for the recipient on pretransplant serology (anti CMV IgG and IgM antibodies). All patients underwent weekly testing for Cytomegalovirus (CMV) antigen (phosphoprotein 65: pp65) or the Digene DNA CMV hybrid capture assay. In 2003 CMV PCR (Roche ^® ^Amplicor, Switzerland) was introduced into clinical practice.

### Data collection and statistical analysis

A database was created using Microsoft excel 5.0. For each patient an individual datasheet was completed, which contained daily body temperature, laboratory results, immunosuppressive and antimicrobial therapy as well as all results from microbiological investigations. Baseline data were prospectively collected and supplemented by retrospectively collected data such as complications, graft loss and death. Statistical analysis was carried out using MS excel and SPSS including Chi-square test and non parametric Mann-Whitney-U or Kruskal-Wallis assay. Data are reported as median with minimum/maximum range, mean ± SD. Survival was calculated using the Kaplan-Meier method. A p-value < 0.05 was considered statistically significant.

## Results

### Patient and graft survival

The median follow up was 3.2 years (range: 0.1–7.8). Overall one- and three-year actuarial patient survival was 96.4% and 94%, respectively. From the 14 deaths, which occurred median 15 months (range 1–74) post transplant, ten occurred with functioning grafts and the remaining four patients lost the pancreatic or renal graft function prior to death. The causes of death were myocardial infarction in five cases, haemorrhage and cerebrovascular insult in one patient each, de novo malignancies in another two patients and infection in the remaining five cases including one pneumonia, one viral infection (HHV6, CMV) and three filamentous fungal infections. No patient died directly related to a blood borne infection.

Kidney one- and three-year survival was calculated to be 94.8% and 93.2%. Seventeen renal grafts (7.7%) were lost after three years. One kidney was lost due to early arterial thrombosis and another due to Polyomavirus-infection. Two patients lost their renal grafts due to chronic dysfunction, in one case associated with drug toxicity and in the other this was due to non compliance. One patient developed infective endocarditis of the tricuspid valve and developed septic arterial graft thrombosis leading to renal graft loss. The patient underwent valve repair but died later on from invasive zygomycosis. The remaining losses were death with functioning graft (DWFG).

Overall one- and three-year pancreas graft survival was 88.5% and 83.8%. Of the 217 pancreas grafts, 182 (84%) are currently functioning without exogenous insulin and normal HbA1c levels at a median follow up of 3.2 (range 0.1–7.8) years. Causes of the 35 pancreas graft losses were irreversible rejection/dysfunction (n = 9), intraabdominal infection (n = 5), arterial thrombosis/infarction (n = 9), arterial bleeding (n = 2), and death with functioning graft (n = 11). Therefore, technical failure (16 grafts) was the most common cause of graft loss with thrombosis accounting for the majority of cases.

### Post operative course

During first hospitalization, a total of 75 rejection episodes in 65 patients (30%) were treated. In 60 instances out of 217 transplants (27%) one or more relaparotomies for surgical complications were necessary within the first post operative (p.o.) year. During the p.o. stay patients developed sub febrile temperatures on 33% and fever over 38 centigrade on 9% of observation days.

### Infectious complications

Within the first three months a total of 208 infectious episodes following 119 transplants were recorded. Another 48 infectious episodes were diagnosed during further follow up. The spectrum of infectious complications is shown in table 2, recurrent infections of the same type were not counted separately. Sepsis was the third most common infectious complication in this series. Only IAI (56 episodes in 47 patients) were diagnosed more frequently and CMV infection and disease (in total 53 patients, six with recurrent episodes). The incidence of the latter significantly declined after change from a preemptive strategy to Ganciclovir prophylaxis for high risk patients.

**Table 1:** demographic and clinical data according to development of sepsis

	no sepsis	sepsis	p-value
n transplants	182	35	
single pancreas	16%	17.4%	n.s.
pancreas retransplant	11%	11.4%	n.s.
ATG single bolus induction	69.2%	62.9%	n.s.
recipient age	41.5 (22.4–62.5)	45.7 (27.2–62.1)	n.s.
recipient age > 55 years	8.8%	17.1%	n.s.
donor age	28 (10–54)	35 (13–51)	0.012
recipient BMI	22.9 (15.1–31.2)	22.7 (18.3–31.5)	n.s.
donor BMI	23.1 (17.3–29.4)	23.9 (18.6–27.1)	n.s.
waiting time to PTx (days)	156 (0–2689)	144 (8–2439)	n.s.
cold ischemia renal graft	11.6 (4–21)	12 (5.8–21.6)	n.s.
cold ischemia pancreas graft	13.1 (5.9–20.6)	13.7 (6.9–19.9)	n.s.
rejection rate	29.7%	31.4%	n.s.
Type II DM	4.4%	17.1%	0.013

**Table 2:** Infectious episodes following 217 pancreatic transplants: high probability of other infections in patients with systemic infection

	Patients	Patients with sepsis	% of patients with sepsis	% of patients without sepsis	significance
**COMMON INFECTIONS**					
CMV infection/disease	53	15	28%	12%	0.004
Intraabdominal infection	47	21	45%	8%	<0.0001
HSV I/II, VZV, HHV6	33	5	15%	16%	0.8
Bloodstream infection	35	35	100%	0%	<0.0001
Wound infection	26	12	46%	12%	<0.0001
Urinary tract infection	22	9	41%	13%	0.003
Respiratory tract infection	9	3	33%	15%	0.2
					
**RARE INFECTIONS**					
Filamentous fungal infection	5	4			
Superficial Candidiasis	2	0			
PTLD	2	0			
Endocarditis	1	1			
Osteomyelitis	1	0			
Gastric ulcer	1	0			
Colitis (C. difficile/Rotavirus)	1	1			
Polyoma Virus nephritis	1	0			

### Monitoring of catheters

**Figure 1:**
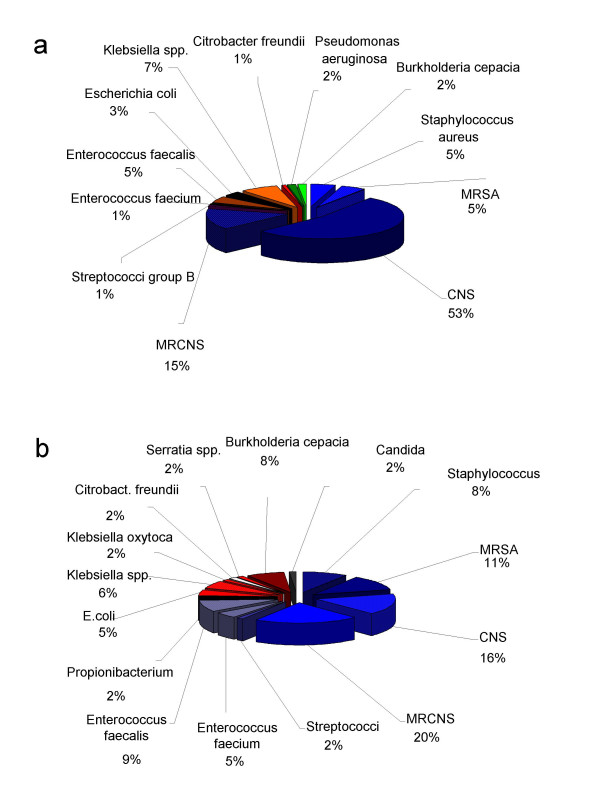
**A** Spectrum of pathogens (n = 121) isolated from catheter tips (n = 322). **B** Spectrum of pathogens (n = 66) isolated from blood cultures (n = 338).

In total 322 tips of central venous catheters were sent for microbiological investigation. Of those 221 (69%) were sterile. There were 19 mixed infections. In total 121 pathogens were cultivated including 103 gram positive cocci, 14 gram negative rods and four non fermentative bacilli. Figure 1a shows the spectrum of pathogens. Large diameter catheters (n = 27) were independently analyzed. None of the nine pulmonary arterial catheters (Swan Ganz) was contaminated, whereas six of nine large pore catheters (multiresistant Staphylococcus aureus 2, coagulase negative Staphylococci 2, multiresistant coagulase negative Staphylococci 1 and multiresistant coagulase negative Staphylococci/Serratia spp. 1) and four of nine Quinton catheters (coagulase negative Staphylococci 3, Enterococcus fecalis 1) were contaminated.

### Results from blood cultures

A total of 338 blood cultures were analyzed (multiple blood cultures taken in the same patient on the same day was considered one event) and 66 pathogens were isolated including 49 Gram positive cocci, ten Gram negative rods and Propionibacterium acnes and Candida albicans in one case each. There were six blood cultures that grew multiple organisms. When looking at the total number of blood cultures (n = 439), 379 were sterile and 60 (14%) showed growth of pathogens. Figure 1b depicts the spectrum of pathogens isolated from blood cultures.

### Sepsis

In total 46 sepsis episodes were diagnosed in 35 patients (16%). Nine patients experienced multiple sepsis episodes with four patients having both IAI with sepsis and line sepsis. In 60% (21 patients) the source of sepsis was IAI, in ten cases the source was a contaminated line and in eight cases other sources were identified. The median onset of sepsis associated with IAI was on day 12 (range 6–45), for line sepsis it was day 12 (range 6–32) and for other causes of septic episodes it was on day 13 (range 1–24) post transplant. The sepsis source in the latter cases were urinary tract (n = 2), graft transmitted (n = 2) and wound infection (n = 5). In total 65 pathogens (IAI sepsis 39, line sepsis 15, others 11) were isolated from blood. Table 3 shows demographic data and the spectrum of organisms according to the source of sepsis. Gram positive cocci accounted for the vast majority of pathogens causing sepsis (50 of the 65 isolates, i.e. 75%). The most commonly isolated single pathogens were coagulase negative staphylococci with 28 isolates of which nine were multiresistant, Staphylococcus aureus accounted for eleven cases (four MRSA) and enterococci for nine cases (one E. faecium). Gram negative rods were cultured in twelve cases. The most common cause of sepsis originating from IAI was graft pancreatitis with 10 cases, followed by peritonitis in five cases and infected ascites in two cases. In one case each an infected peripancreatic seroma, an intraabdominal hematoma, graft necrosis due to portal vein thrombosis and a leak at the distal stapler line of the duodenal segment were the source of sepsis. The spectrum of pathogens changed during the study period with intensified perioperative antibiotic prophylaxis. Whereas for the first 112 cases polymicrobial infection including Gram negative rods and Gram positive cocci was present in eight of 12 cases, during the last 105 cases six of nine sepsis cases originating from IAI were monomicrobial with CNS accounting for four episodes. This includes also the patient with the duodenal leak. In terms of management of IAI, 18 of the 21 patients with IAI underwent relaparotomie; the remaining three percutaneous drainage. In addition to the above mentioned intraabdominal pathology, intraoperative findings were infected hematoma (n = 6), which were associated with pancreatitis in four cases and a thrombosed graft in one case, wound infection (n = 12), and wound dehiscence (n = 3). During these 18 interventions four grafts had to be removed.

**Table 3:** Spectrum of pathogens and clinical and demographic data according to the source of sepsis

**SEPSIS**	**IAI**	**line**	**others**	**total**
**n patients**	21	10	8	39*
**n episodes**	26	11	9	46
**mixed infection**	12	5	2	19
**n pathogens**	39	15	11	65
				
**Gram positive cocci**	29	12	9	50
**Coagulase negative Staphylococci**	10	4	5	19
**MR Coagulase negative Staphylococci**	7	2	0	9
**Staphylococcus aureus**	3	4	0	7
**MR Staphylococcus aureus**	2	1	1	4
**Streptococci**	1	1	0	2
**Enterococcus faecalis**	5	0	3	8
**Enterococcus faecium**	1	0	0	1
**Gram negative rods**	8	2	2	12
**Escherichia coli**	2	0	0	2
**Klebsiella spp.**	3	2	1	6
**Enterobacter spp.**	2	0	1	3
**Serratia marcescens**	1	0	0	1
**Propionibacterium spp.**	1	0	0	1
**Pseudomonas aeruginosa**	1	1	0	2
				
**Graft loss**	2	0	1	3
**onset post transplant (range)**				
**median**	12	12	13	12
**min**	6	6	1	1
**max**	45	32	24	45
				
*4 patients had line sepsis and IAI with sepsis				
MR: multi resistant				

A significant reduction in the rejection rate was observed during the study period from 41% for the first 112 cases to 18% for the following 105 cases. Concomitantly the rate of blood stream infection rose from 13% to 19%. The difference did not reach statistic significance.

Out of the 35 patients with sepsis 33 (94.3%) developed also other infectious complications, whereas 52.7% of the 182 patients who had no sepsis had other infections (p < 0.0001). A total of 15 patients had sepsis and CMV infection/disease. In eleven of the 15 cases, sepsis onset proceeded CMV infection/disease, with a median interval of 20 (range 2–109) days. Ganciclovir prophylaxis for all CMV mismatched transplants in the last 105 cases had a major impact on the incidence of CMV infection/disease. Whereas 12 of 15 patients who developed sepsis during the early period (first 112 cases) also had CMV infection/disease, only three of 20 patients of the later cohort developed sepsis and CMV infection/disease.

**Figure 2:**
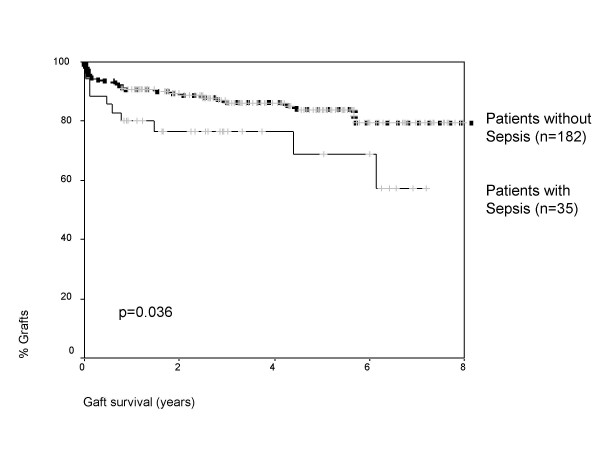
Graft survival according to the development of boodstream infection.

Table 1 shows demographic and clinical data for patients with and without sepsis. Donor age (35 versus 28 years) and Type II diabetes mellitus (17.1% versus 4.4%) were found to be significant risk factors for the development of sepsis, whereas recipient age, rejection rate and ischemic time had no impact. Figure 2 shows pancreas graft survival for patients with and without sepsis. Patients with blood borne infections had a one/two and five year graft survival of 80%/76.5% and 68.9% versus 90%/89.4% and 83.9% for those without sepsis (p = 0.036). Also the renal graft survival was significantly worse in patients who developed bloodstream infection with one/two and five year graft survival of 92%/92% and 78% versus 96%/95% and 94% for those without sepsis (p = 0.033). Sepsis had no significant impact on patient survival and was in patients who developed bloodstream infection at one/two and five years 96.2%/96.2% and 90.2% versus 96.5%/95.8% and 93% for those without sepsis (p = 0.6).

## Discussions and conclusion

This study comprising a large number of patients demonstrates that blood stream infection is a significant complication in pancreas recipients. In our series 35 patients (16%) developed systemic infection. Sepsis was the third most common infection in this series. Most commonly the source was IAI, however, in eleven cases the source was a contaminated central venous line. Rare sources were urinary tract infection and wound infection. Of note, in two cases a contaminated graft resulted in systemic infection. The most common single pathogen was Staphylococcus epidermidis. We were able to identify advanced donor and recipient age and type II diabetes mellitus to be risk factors for the development of sepsis. Blood stream infection had a significant impact on pancreatic and renal graft survival.

Pancreas transplantation has become widely accepted as therapy for patients with end stage diabetic nephropathy [2]. According to the International Pancreas Transplant Registry report, the technical failure rates exceed the immunological failure rates [1]. In our series of 217 consecutive enteric drained pancreas transplants using ATG induction, Tacrolimus, MMF and steroids prophylactic immunosuppression one – year patient, pancreas and kidney graft survival was 94%, 83% and 90%. Overall, 30% of patients had to be treated for rejection. The rejection rate in this series declined from 41% in earlier cases to 18%, but at the same time the incidence of sepsis increased from 13% to 19%. For the usage of Sirolimus no increased infection rate or sepsis rate could be observed, however, the number of patients receiving this new agent in this series is too small to draw final conclusions.

Infection remains a common complication following solid organ transplantation [5,15,17]. Although many of these infections are well controllable due to rapid detection and highly active antimicrobial agents, some of them remain a significant threat for the patient. In pancreas recipients postoperative infections are reported to occur in 50–100% [7,10,18-21]. Bacterial infections, especially systemic infection, remain a significant source of morbidity and mortality during the early postoperative period [6,8]. In our series the overall infection rate was 59% with sepsis being the third most common infection. Similar to previously published data, sepsis originating from IAI was associated with significantly increased hospitalization and costs and a poorer graft survival [22-25]. The most common source of systemic infections in our series was intraabdominal infection. In total 45 out of the 217 patients (20.7%) developed IAI and of 138 organisms were cultured including Gram positive cocci (n = 69), Gram positive rods (n = 6), Gram negative rods (n = 35), non fermentative bacilli (n = 13), anaerobes (n = 7), fungi (n = 8). Most commonly IAI was associated with pancreatitis. Only one case, which led to sepsis, was associated with an anastomotic leakage. Ten of 15 patients transplanted during the early period had multiple episodes of sepsis. Of these, nine were associated with ongoing IAI. During the later period only six of 20 patients had multiple episodes of sepsis. This included four patients with ongoing IAI. In the cases of ongoing IAI causing sepsis antibiotic therapy had to be adapted several times due to a shift in the spectrum of isolated pathogens. Severe early post-transplant IAI was a significant risk factor for graft loss. Line sepsis was the second most common systemic infection whereas other sources for sepsis are rare. Notably, five cases derived from wound infection. Wound infection in pancreas recipients with long standing diabetes and angiopathy who receive steroids has a tendency of delayed healing. This might explain the high frequency of wound infections that spread to the bloodstream. If the new immunosuppressive agents Sirolimus or Everolimus are added even slower healing may be expected [26,27].

Coagulase negative staphylococci were the predominant pathogens causing sepsis in our series regardless of the origin. One third of these isolates were multiresistant, and out of nine isolates of Staphylococcus aureus four were multiresistant. Nevertheless, this fact does not justify to add a glycopeptide to the prophylactic regimen. However, if sepsis is suspected, a glycopeptide should be included in the empiric therapy until blood cultures are reported. No case of candidemia was observed; however, in eight cases of IAI, candida was isolated from the abdominal cavity. Notably for the last 168 cases fluconazole prophylaxis was used. Per protocol in the case of IAI we include an antifungal agent in the empiric antimicrobial regimen. After a first case of Candida krusei IAI we use Caspofungin (70 mg starting dose followed by 50 daily) in these cases. The empiric antibacterial combination for IAI consists preferably of a Glycopeptide in combination with a Carbapenem. For prevention of line sepsis, intravascular catheters are removed at the earliest possible time point. This may be particularly important for large diameter central venous lines. For prevention of sepsis associated with urinary tract infection, early removal of the catheter was performed. In our series only two cases of sepsis associated with UTI were diagnosed. The incidence of urinary tract infection in our series was 10% with only 4% during the early period, however 17% during the later period. Possibly, the intensified immunosuppression and changing patient demographics such as increased recipient age and transplantation of patients suffering from type II DM could explain this recent increased frequency.

In summary, combined pancreas-kidney transplantation with systemic venous/enteric exocrine drainage under Tacrolimus/MMF immunosuppression using a short course of high dose ATG induction results in a low rate of immunological graft losses (2.1%/yr at our institution). However, there remains considerable surgical morbidity with a 6.5% technical failure rate within the first post operative year. Infection remains the most common complication and the most common cause of graft loss and death. Prevention of systemic infections targets for prevention of IAI as first step as IAI is the most common source. Sterile handling of central venous lines and removal at the earliest possible time point are further steps. Importantly, two cases in this series derived from urinary tract infection and another two cases were transmitted by the graft. Further improvements in pancreas preservation, improvement in infection prophylaxis and better control of intraabdominal infectious complications will be needed to reduce the incidence of bloodstream infections and further improve results following pancreas transplantation [6,28,29].

## Pre-publication history

The pre-publication history for this paper can be accessed here:


